# Coronary microcirculatory dysfunction after percutaneous coronary intervention: pathogenesis, diagnosis, and therapeutic strategies

**DOI:** 10.3389/fcvm.2025.1618242

**Published:** 2025-11-13

**Authors:** Jin BoYuan, Wang TingTing, Hua ChengJun, Han XinYi, Chen YuShan

**Affiliations:** 1 National Regional (Traditional Chinese Medicine) Cardiovascular Diagnosis and Treatment Center, Heart Center of the First Affiliated Hospital of Henan University of Traditional Chinese Medicine, Zhengzhou, Henan, China; 2 Emergency Department, First Affiliated Hospital of Henan University of Traditional Chinese Medicine, Zhengzhou, Henan, China

**Keywords:** coronary microcirculation disorder, percutaneous coronary intervention therapy, oxidative stress, inflammation, diagnosis and treatment

## Abstract

Coronary microvascular dysfunction (CMD) can lead to a variety of severe adverse cardiovascular events. CMD represents the primary cause of recurrent angina pectoris following percutaneous coronary intervention (PCI). The etiology of post-PCI CMD is complex and largely occult, which significantly impairs the therapeutic efficacy of PCI. This article reviews the physiological functions of the coronary microcirculation, as well as the latest research progress on the pathogenesis, diagnosis, and treatment of CMD after PCI. Finally, it highlights the scientific issues that urgently need to be addressed regarding CMD after PCI and proposes future research directions, with the aim of providing forward-looking insights for the prevention and treatment of CMD after PCI in the future.

## 1 Introduction

Ischemic heart disease is the leading cause of death worldwide (1, 2), with 18 million people dying from cardiovascular diseases each year. It is projected that by 2030, the number of people dying from cardiovascular-related diseases globally each year will reach 24 million, averaging over 66,000 people per day, and the total global cost will exceed 1 trillion US dollars (1). Due to its advantages such as safety, minimal invasiveness, and high efficiency, PCI has become the most important treatment method for opening diseased blood vessels and restoring myocardial blood supply (3). However, after PCI, some patients experience recurrent angina pectoris. Studies have shown that the incidence of recurrent angina pectoris after PCI is as high as 18%–34% (4). Recurrent angina pectoris arises from multiple causes, with coronary microvascular dysfunction (CMD) being the most prevalent (5). Currently, direct visualization of coronary microvascular perfusion remains unachievable, rendering the diagnosis of CMD considerably challenging. Commonly, functional parameters such as coronary flow reserve (CFR), index of microcirculatory resistance (IMR), and fractional flow reserve (FFR) are used to indirectly diagnose CMD. There are numerous causes of CMD after PCI, which can be affected by various factors before, during, and after PCI. In response to these mechanisms, the incidence of CMD after PCI can be reduced and the prognosis of patients can be improved through measures such as controlling risk factors and implementing interventions before, during, and after PCI. This article first summarizes the pathophysiological mechanisms of CMD and reviews the specific mechanisms by which factors before, during, and after PCI lead to the occurrence of CMD. Subsequently, we summarize the invasive and non-invasive diagnostic methods for CMD and compare the advantages and disadvantages of various diagnostic methods. In addition, we also introduce the latest treatment methods. Finally, we put forward the scientific issues that urgently need to be resolved for CMD after PCI and outline the future research directions. It is anticipated that this review will offer valuable insights for the clinical management of post-PCI CMD.

## 2 Pathophysiology of coronary microcirculation

**Figure 1:**
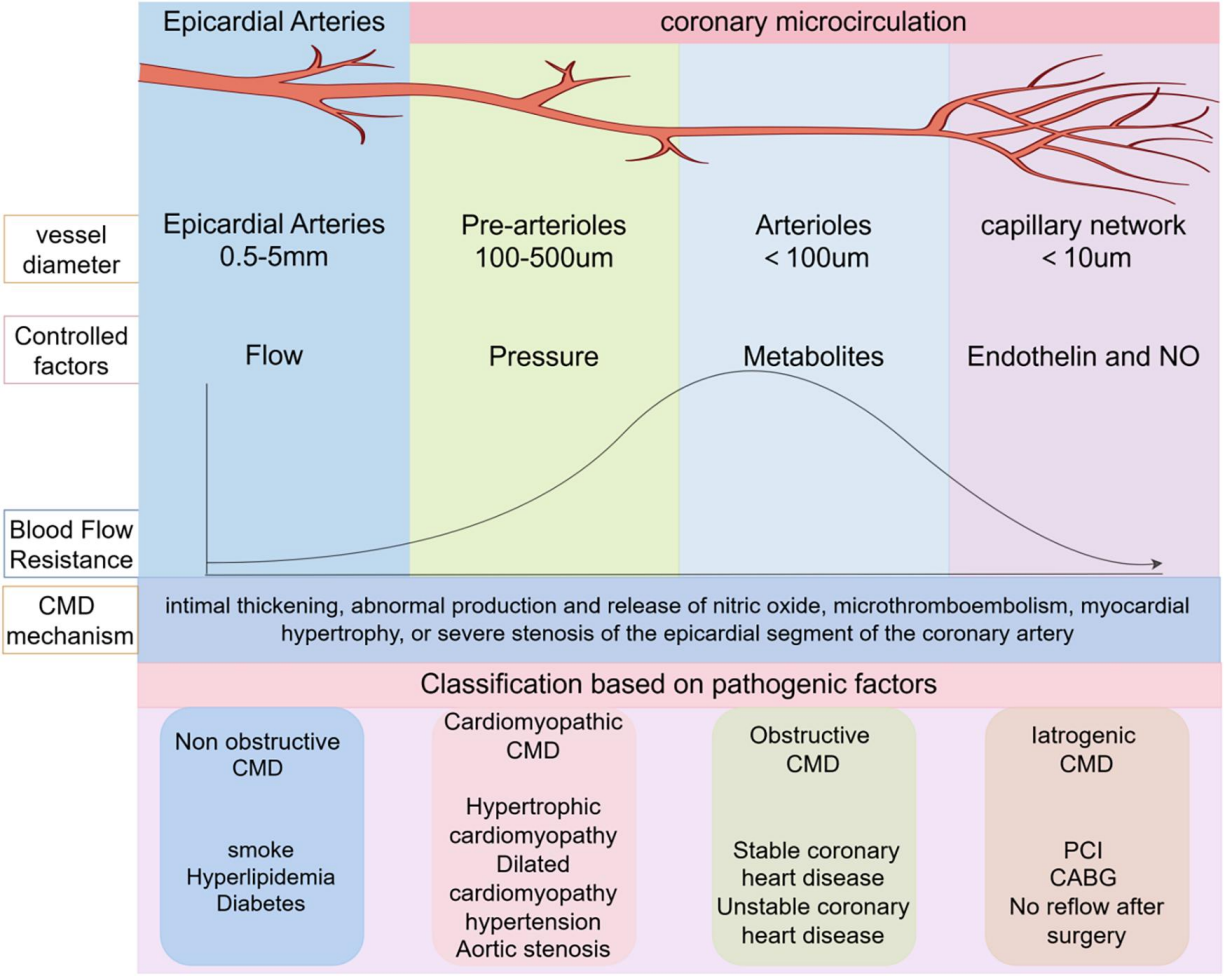
Coronary artery anatomy and CMD classification. PCI, percutaneous coronary intervention; CMD, coronary microvascular dysfunction; CABG, coronary artery bypass grafting.

The coronary artery system of the heart is divided into the epicardial coronary artery segment and the coronary microcirculation segment. The epicardial artery segment has a lumen diameter ranging from 0.5 to 5 mm, primarily responsible for blood transportation. It is the main site prone to atherosclerosis and can be visualized by coronary angiography. The coronary microcirculation segment can be further classified into pre-arterioles and arterioles (6). Pre-arterioles, with a lumen diameter of 0.1–0.5 mm, are pressure-sensitive arteries. When the blood supply from the epicardial artery segment changes, the diameter of pre-arterioles also adjusts, thereby stabilizing the blood supply to the myocardium. Arterioles, with a diameter less than 0.1 mm, are mainly influenced by local myocardial metabolites. When local myocardial metabolites accumulate excessively, the diameter of arterioles expands, reducing coronary vascular resistance and increasing myocardial blood supply (7). Pre-arterioles and arterioles serve as the primary resistance vessels of the coronary arteries and the sites of myocardial metabolism. They play a crucial role in regulating coronary blood flow (CBF). At rest, myocardial oxygen uptake is already near its maximum capacity; therefore, the potential for enhancing myocardial oxygen delivery relies almost entirely on increased coronary blood flow (8) (Figure 1).

Pre-arterioles and arterioles are not detectable via coronary angiography, presenting substantial obstacles to the diagnosis and management of coronary microcirculatory disorders. Clinically, coronary flow reserve (CFR), index of microcirculatory resistance (IMR), and fractional flow reserve (FFR) are important indicators reflecting the function of coronary microvessels (9). Under physiological conditions, factors such as blood pressure, oxygen content, and metabolite accumulation can regulate the constriction and dilation of coronary microvessels, thereby modulating CFR. However, under pathological conditions, due to factors such as intimal thickening, abnormal production and release of nitric oxide, microthrombus embolism, myocardial hypertrophy, or severe stenosis of the epicardial coronary artery segment, the function of coronary microvessels is impaired, leading to the occurrence of coronary microvascular dysfunction (CMD). CMD manifests as clinical symptoms such as chest tightness, angina pectoris, exertional dyspnea, and decreased exercise tolerance (10). Based on clinical characteristics and causative factors, coronary microcirculation diseases can be classified into four categories: (1) Non-obstructive CMD, commonly seen in individuals with smoking habits, hyperlipidemia, and diabetes; (2) Cardiomyopathic CMD, often associated with hypertrophic cardiomyopathy, dilated cardiomyopathy, hypertension, and aortic stenosis; (3) Obstructive CMD, typically found in patients with stable and unstable coronary heart disease; (4) Iatrogenic CMD, observed in patients with no-reflow phenomenon after percutaneous coronary intervention (PCI) or coronary artery bypass grafting (CABG) (11). Clinical practice and literature reviews indicate that the incidence of recurrent angina pectoris following PCI ranges from 18% to 34%. Among these recurrent cases, iatrogenic CMD accounts for a significant proportion, severely affecting the doctor-patient relationship and patient prognosis (4). Therefore, exploring the pathogenesis, diagnosis, and treatment strategies of CMD after PCI is of great significance.

## 3 PCI and CMD

**Figure 2:**
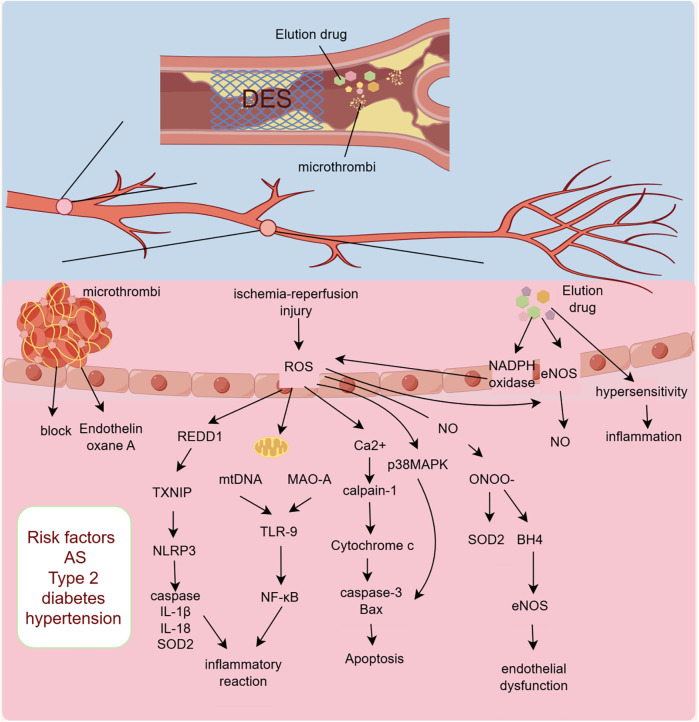
Mechanism of CMD occurrence after PCI. DES, drug-eluting stents; ROS, reactive oxygen species; REDD1, DNA damage response regulator 1; TXNIP, thioredoxin-interacting protein; NLRP3, NOD-like receptor thermal protein domain associated protein 3; SOD2, superoxide dismutase 2; mtDNA, mitochondrial DNA; MAO-A, monoamine oxidase A; TLR-9, toll-like receptor 9; NF-κB, nuclear factor kappa-B; ONOO-, peroxynitrite; BH4, tetrahydrobiopterin; eNO, endothelial nitric oxide synthase; AS, Atherosclerosis.

PCI, which can rapidly unclog narrowed or occluded lumens and restore blood flow, is the preferred treatment for opening diseased blood vessels in patients with STEMI. However, CMD—also referred to as the “no-reflow phenomenon”—still occurs in a subset of patients following PCI (12). The occurrence of CMD after PCI is associated with multiple factors. First, patients with pre-PCI risk factors for CMD are more prone to developing severe myocardial ischemia symptoms after the procedure. Secondly, the operations during PCI and the eluting drugs of PCI stents can also promote the occurrence of CMD, affecting patient prognosis. Finally, after PCI, the rapidly restored coronary blood flow can lead to ultrastructural and functional changes at the microvascular level, including platelet aggregation, microvascular spasm, inflammatory response, endothelial cell ischemia, and reperfusion injury, (Figure 2; Table 1).

**Table 1:** Pathophysiological mechanisms of CMD after PCI.

Period	Category	Specific mechanism	Key molecules/processes	Pathological consequences
Pre-PCI	Vascular Structural & Functional Abnormalities	Epicardial artery stenosis caused by atherosclerosis	Low shear stress	Increased endothelial permeability to ox-LDL and inflammatory cells, inducing endothelial cell apoptosis and inflammation (13–18)
	Vascular Structural & Functional Abnormalities	Hypertension	Myocardial hypertrophy, small artery remodeling, over-activation of the RAAS, increased sympathetic nervous activity	Microvascular compression and rarefaction, increased microvascular resistance, endothelial dysfunction (25–28)
	Metabolic & Endocrine Abnormalities	Type 2 diabetes	Hyperglycemia, AGEs, elevated expression of miRNAs in the miR-30d family, oxidative stress, CAN	Endothelial dysfunction, inhibition of angiogenesis, reduced myocardial microvascular density, impaired vasodilation regulation (19–24)
Intra-PCI	Device & Procedural Injury	Local toxicity of drug-eluting stents (DES)	Sirolimus and other stent-eluting drugs, enhanced expression of NADPH oxidase, stimulation of mitochondrial free radicals, reduced eNOS phosphorylation	Local endothelial dysfunction and apoptosis, delayed healing, excessive release of oxygen free radicals reducing NO bioavailability (30–38)
	Embolization & Mechanical Obstruction	Dislodgement of microthrombi or plaque debris	Platelet aggregation, activation of the coagulation system	Mechanical obstruction of distal microvessels, focal myocardial infarction, release of vasoconstrictors (e.g., endothelin, thromboxane A2) (39–42)
Post-PCI	Oxidative Stress	eNOS uncoupling	BH4, excessive production of ROS	Reduced NO synthesis, formation of superoxide anions and peroxynitrite (ONOO^−^) (50–53, 56)
	Oxidative Stress	NO consumption and signaling inhibition	Reaction of O₂⁻ with NO to form ONOO⁻, inhibition of SOD2 activity	Loss of NO-mediated vasodilation, impaired antioxidant defense system, vicious cycle (51, 52)
	Inflammatory Response	NLRP3 inflammasome activation	ROS/REDD1/TXNIP pathway, mtDNA leakage activating TLR9/NF-*κ*B	Caspase-1 activation, release of pro-inflammatory cytokines IL-1β and IL-18, exacerbating inflammation and pyroptosis (57–61)
	Inflammatory Response	Systemic inflammatory marker	hs-CRP	Complement activation, expansion of infarct area, positive correlation with microvascular resistance (66, 67)
	Apoptosis & Cell Death	Mitochondrial apoptotic pathway activation	ROS-induced release of cytochrome c and AIF, Ca²⁺ influx activating calpain-1 and caspase	Apoptosis of endothelial cells and vascular smooth muscle cells, disruption of microvascular integrity (62–65)
	Leukocyte & Platelet Activation	Platelet aggregation and adhesion	Loss of endothelial anticoagulant function in the reperfused area	Microthrombosis, further obstruction of microvessels (6, 107)
	Leukocyte & Platelet Activation	Neutrophil infiltration	Release of chemokines from ischemic myocardium	Capillary obstruction, release of ROS and proteases, causing “bystander” damage (124)

### 3.1 Mechanisms of CMD

All stages (early, middle, and late) of atherosclerosis can affect the coronary microcirculation and induce the occurrence of CMD. Even in patients with only risk factors for coronary heart disease (such as diabetes and hypertension), CFR is impaired (13).

#### 3.1.1 Atherosclerosis

Coronary artery stenosis in the epicardial segment caused by atherosclerosis can lead to CMD. When the epicardial coronary artery is stenosed, the reduction in coronary perfusion pressure leads to changes in shear stress. Shear stress can affect the morphology, intimal proliferation, differentiation, metabolism, and cell signaling of endothelial cells (14). Under physiological conditions, changes in fluid shear stress can control the contraction and dilation of blood vessels by influencing the release of NO from endothelial cells (15). In this process, Kruppel-like factor 2 (KLF2) in endothelial cells plays a critical role. Under normal physiological shear stress, KLF2 is activated, which in turn upregulates the expression of nitric oxide synthase (eNOS) and inhibits the production of adhesion molecules. In contrast, excessive reduction of shear stress leads to downregulated KLF2 expression, increasing the exposure of endothelial adhesion molecules (such as ICAM-1 and VCAM-1). This accelerates the recruitment and infiltration of monocytes, thereby initiating the microvascular inflammatory response (16).

Moreover, when the fluid shear stress is excessively reduced, the permeability of endothelial cells to ox-LDL and inflammatory cells increases, inducing endothelial cell apoptosis and the progression of inflammation (17, 18). After ox-LDL enters endothelial cells, it can activate the toll-like receptor 4 (TLR4)/myeloid differentiation factor 88 (MyD88) signaling pathway, promoting the nuclear translocation of nuclear factor κB (NF-κB). This regulates the secretion of pro-inflammatory cytokines such as tumor necrosis factor-α (TNF-α) and interleukin-6 (IL-6), further exacerbating microvascular endothelial injury and inflammatory infiltration (19). Meanwhile, ox-LDL can also inhibit the proliferation and migration capabilities of endothelial progenitor cells, impairing the self-repair function of microvessels (20).

When the fluid shear stress is increased, it can affect endothelial function through mechanical and biochemical means. The mechanical effect is to induce endothelial cell exfoliation and trigger endothelial cell death, while the biochemical effects include increasing NO production and affecting the activation of growth factors and von Willebrand factor (21). High shear stress can additionally activate the mitogen-activated protein kinase (MAPK) pathway, promoting the abnormal proliferation of vascular smooth muscle cells and their migration to the intima. This results in thickening of the microvascular wall, luminal stenosis, and accelerated progression of CMD (22).

#### 3.1.2 Type 2 diabetes

Type 2 diabetes and the pre-diabetic state of type 2 diabetes can significantly increase the incidence of CMD. Firstly, endothelial dysfunction and its associated adverse consequences are widely recognized as results of diabetes (23). A long-term hyperglycemic environment damages mitochondrial function within endothelial cells, thereby inhibiting angiogenesis and leading to oxidative stress and metabolic disorders (24). Impaired mitochondrial function leads to abnormalities in the electron transport chain, triggering the massive production of reactive oxygen species (ROS). ROS can reduce the activity of eNOS through oxidative modification, decreasing NO synthesis. Simultaneously, it activates the p38 mitogen-activated protein kinase (p38 MAPK) and c-Jun N-terminal kinase (JNK) signaling pathways, accelerating endothelial cell apoptosis (25).

Secondly, studies have shown that in the cardiac tissues of diabetic mice, the expression of miRNAs in the miR-30d family is significantly elevated, resulting in reduced myocardial microvascular density and CMD (26). miR-30d can directly target and regulate the expression of vascular endothelial growth factor (VEGF). As a key factor in maintaining microvascular integrity and promoting angiogenesis, reduced VEGF expression hinders endothelial cell proliferation, impairs vascular formation capacity, and ultimately leads to a decrease in microvascular density (27). Additionally, hyperglycemia promotes the formation of advanced glycation end products (AGEs), which exacerbate oxidative stress through the hexosamine, polyol, and protein kinase C pathways, leading to cellular and tissue damage (28). When the polyol pathway is activated, aldose reductase converts glucose into sorbitol. Due to sorbitol's poor permeability across cell membranes, it accumulates intracellularly, increasing intracellular osmotic pressure and inducing endothelial cell edema (29). After activation of protein kinase C, it can inhibit eNOS activity and promote the production of vasoconstrictive substances such as thromboxane A2 and angiotensin II (Ang II), resulting in microvascular vasomotor dysfunction. Additionally, it can stimulate the release of platelet-activating factor, inducing a hypercoagulable state and microthrombus formation (29).

AGEs can also activate signaling pathways within endothelial cells, triggering apoptosis, inflammatory responses, and microthrombosis (30). The specific mechanism involves the binding of AGEs to their receptor RAGE, which continuously activates the ROS generation system and amplifies inflammatory signaling pathways. This promotes the release of pro-inflammatory cytokines such as TNF-α and IL-6, forming a vicious cycle of oxidative stress and chronic inflammation that disrupts microvascular homeostasis (31). Meanwhile, this signaling pathway can also activate plasminogen activator inhibitor-1, inhibiting fibrinolysis and accelerating microthrombus formation (32). Cardiovascular autonomic neuropathy (CAN) is a complication of diabetes, and it can affect the autonomic control of the diameter of pre-arterioles, thereby inducing CMD (33). The autonomic nerve imbalance caused by cardiovascular autonomic neuropathy (CAN) further leads to abnormally increased sympathetic nerve excitability. Through the release of norepinephrine to activate α1-adrenergic receptors, it induces sustained contraction of microvascular smooth muscle. At the same time, it inhibits NO release from endothelial cells, further exacerbating increased microvascular resistance and insufficient perfusion (34).

#### 3.1.3 Hypertension

Hypertension causes damage to small arterioles earlier than other arteries and is a significant high-risk factor for CMD. Firstly, hypertension directly damages microvessels, leading to a reduction in their number and a narrowing of their diameter, thereby increasing cardiac afterload and reducing the myocardial perfusion ratio (35). Chronic exposure to high pressure activates the mechanosensitive ion channel TRPV4 in microvascular endothelial cells, triggering calcium ion influx. This further activates the calcineurin (CaN)/nuclear factor of activated T-cells (NFAT) signaling pathway, promoting the endothelial-to-mesenchymal transition of endothelial cells. This process can lead to fibrosis of the microvascular wall and luminal stenosis (36). Secondly, hypertension can cause myocardial hypertrophy; the increased oxygen demand of excessively thickened myocardium can compress microvessels and increase microvascular resistance (37). During myocardial hypertrophy, hypertrophic cardiomyocytes secrete transforming growth factor-β1 (TGF-β1). By activating the Smad2/3 signaling pathway, TGF-β1 promotes the proliferation of perivascular fibroblasts and collagen deposition. This results in compression and deformation of microvessels, impeding blood perfusion (38).

Additionally, increased sympathetic nervous activity in hypertensive patients can lead to cardiac remodeling and excessive constriction of pre-arterioles, resulting in increased microvascular resistance (39). Hypertension can also damage endothelial cells by reducing the release of nitric oxide (NO), promoting over-activation of the renin-angiotensin-aldosterone system (RAAS), increasing homocysteine (Hcy) levels, and enhancing inflammatory responses (40). Following activation of the RAAS, Ang II binds to the AT1 receptor, which activates NADPH oxidase to generate large amounts of reactive oxygen species (ROS). ROS oxidatively modifies endothelial nitric oxide synthase (eNOS), causing its uncoupling and reducing NO synthesis. Meanwhile, Ang II also promotes the activation of NF-κB, upregulating the expression of adhesion molecules and pro-inflammatory cytokines, thereby exacerbating microvascular inflammatory injury (41).Elevated homocysteine (Hcy) levels induce microvascular endothelial dysfunction and increase the risk of thrombosis by inhibiting eNOS activity, promoting ROS production, and activating coagulation factor VIII (42). Additionally, in the context of hypertension, reduced erythrocyte deformability and increased erythrocyte aggregation lead to elevated blood viscosity, which further impairs microcirculation and accelerates microvascular damage (43).

### 3.2 Mechanisms leading to CMD during PCI

In addition to the pre-PCI risk factors and atherosclerosis, the PCI procedure itself can also lead to the occurrence of CMD. Firstly, drug-eluting stents (DES) used during PCI can cause significant endothelial damage and inflammatory responses. Moreover, the PCI procedure can lead to plaque rupture or microthrombus formation, and these microthrombi can obstruct downstream microvessels and induce perivascular inflammatory reactions (44).

Numerous studies have shown that endothelial dysfunction is more severe after the implantation of DES compared to bare-metal stents (BMS). This evidence suggests that the drugs eluted from DES are likely to cause endothelial damage and the occurrence of CMD (45–49). Drugs eluted from DES are typically fully released within one month, yet the impairment of endothelial function can persist for an extended period. Additionally, studies have shown that the stent-eluting agents can cause microvascular dysfunction in distal organs such as the liver and kidneys (50). Therefore, investigating the causes of endothelial dysfunction induced by stent-eluting agents has become an important research direction. It is currently believed that DES leads to endothelial dysfunction by promoting the production and release of superoxide. The eluting agents can enhance the expression of NADPH oxidase and stimulate the release of mitochondrial free radicals; the excessive release of oxygen free radicals can directly damage the mitochondrial function of vascular endothelium, creating a vicious cycle that ultimately leads to endothelial cell apoptosis (51). Furthermore, other experiments have shown that the stent-eluting agent sirolimus can increase protein kinase C-mediated phosphorylation of endothelial nitric oxide synthase, leading to reduced production of vascular nitric oxide (NO) and endothelial dysfunction (52). There is also a view that DES leads to endothelial dysfunction due to acute or delayed hypersensitivity reactions. As foreign materials, stents can be attacked by the host immune system, inducing vascular inflammatory reactions and causing adverse outcomes (53).

Microvascular obstruction is the first step in the initiation of microcirculatory dysfunction. In patients with acute myocardial infarction (AMI), coronary microthrombi mainly originate from the shedding of vulnerable atherosclerotic plaques and mural thrombi, causing microembolism and activating the coagulation system, which is often underdiagnosed and underestimated clinically (54). During PCI, some microthrombi may dislodge and block downstream microvessels (55). Myocardial focal infarction caused by microthrombi is difficult to identify by routine diagnostic methods in a short time. The site of myocardial infarction may progress from the infarction core to the epicardium, and the likelihood of re-infarction in non-infarcted areas significantly increases (56). PCI-related microthrombus obstruction not only leads to focal myocardial infarction but also promotes the release of vasoconstrictors and coagulation substances, such as endothelin and thromboxane A2, further aggravating local tissue damage (57).

### 3.3 Mechanisms leading to CMD after PCI

After PCI rapidly reopens the diseased vessel, the downstream vessels and tissues in the myocardial infarction area undergo severe ischemia-reperfusion injury, leading to further disruption of the microcirculation. During this process, oxidative stress is the core pathological process; it damages endothelial cell function by reducing the synthesis, release, and bioavailability of nitric oxide (NO) (58). Additionally, oxidative stress can interact with inflammatory responses, causing further damage to or even death of endothelial cells. The death of endothelial cells, due to the loss of their barrier and anticoagulant functions, can lead to microthrombosis and microvascular obstruction, reducing microvascular density and ultimately resulting in severe CMD (59–63).

The synthesis and release of NO by endothelial cells are key regulators of endothelium-dependent vasodilation. Moreover, NO has functions in inhibiting platelet aggregation and adhesion, preventing thrombosis, and regulating the proliferation of vascular smooth muscle cells (64). The synthesis of NO in endothelial cells depends on endothelial nitric oxide synthase (eNOS) in its coupled form. ROS can significantly reduce eNOS expression and phosphorylation, thereby decreasing NO production (65). Furthermore, superoxide anions generated by oxidative stress during reperfusion can react with NO to form peroxynitrite (ONOO-), which induces nitrosative modification of myocardial proteins, leading to myocardial damage (66). This reaction can also competitively inhibit the activity of superoxide dismutase 2 (SOD2) with ROS, reducing ROS clearance, creating a vicious cycle that continuously lowers NO levels in endothelial cells (67). Peroxynitrite (ONOO-) also induces the oxidation of tetrahydrobiopterin (BH4), a cofactor for eNOS, leading to eNOS uncoupling and its conversion into a pro-oxidant, which further stimulates ROS production, causing cellular damage (68). ROS can also indirectly affect NO production through multiple pathways: by activating the phosphorylation of the c-Jun N-terminal kinase (JNK)/p38 MAPK pathway, inhibiting eNOS expression and activity (69); by decreasing the expression of asymmetric dimethylarginine (ADMA) and increasing the expression and activity of dimethylarginine dimethylaminohydrolase II (DDAH II), which inhibits eNOS phosphorylation (70); and by inhibiting nicotinamide nucleotide transhydrogenase (NNT) activity, which also inhibits eNOS phosphorylation (71).

Reactive oxygen species (ROS) can activate DNA damage response regulator 1 (REDD1), which is an inflammation initiator. REDD1 can activate downstream thioredoxin-interacting protein (TXNIP), a ROS-sensitive protein that can directly bind to nucleotide-binding oligomerization NOD-like receptor thermal protein domain associated protein 3 (NLRP3) inflammasome and promote its activation (72). The integrated stress response of endothelial cells activated by ROS also participates in the activation of NLRP3 (73). NLRP3 induces cellular inflammatory responses and apoptosis by recruiting and activating apoptotic factor caspase and pro-inflammatory cytokines IL-1β and IL-18, while also inhibiting the activity of superoxide dismutase 2 (SOD2), leading to severe cellular damage (74).

Additionally, ROS can cause mitochondrial DNA (mtDNA) to leak into the cytoplasm, activating toll-like receptor 9 (TLR-9), which recognizes unmethylated CpG dinucleotides within cells. TLR-9 induces the activation and translocation of NF-κB, leading to an inflammatory response (75). ROS can also activate monoamine oxidase A (MAO-A) in the inner mitochondrial membrane, which catalyzes the degradation of serotonin and is also involved in the activation of TLR-9 (76). Furthermore, ROS can cause the release of Ca2+ from the endoplasmic reticulum into the cytoplasm, activating calpain-1. Calpain-1 induces the release of cytochrome c from the mitochondria into the cytoplasm, where it activates caspase-3 and promotes the activation and translocation of the pro-apoptotic protein Bax to the mitochondria, leading to apoptosis (77). ROS-activated p38 mitogen-activated protein kinase (p38MAPK) also participates in the activation of Bax and caspase-3 (78).

Studies have found that ROS can directly induce the release of cytochrome c and apoptosis-inducing factor (AIF) from the mitochondria into the cytoplasm, triggering inflammatory responses, and simultaneously activating the mitochondrial apoptotic pathway through caspase-9, leading to cell apoptosis (79, 80). Furthermore, research indicates that high-sensitivity C-reactive protein (hs-CRP), an important inflammatory marker, is closely associated with CMD. Hs-CRP is involved in the pathophysiological changes following myocardial infarction, can activate complement, further promote inflammatory responses, and thus expand the infarct area. Studies have shown that patients with high hs-CRP levels in acute myocardial infarction generally have a poorer prognosis (81).

## 4 Diagnostic techniques

Currently, there is no method available for directly observing the structure of the coronary microcirculation. Therefore, existing evaluation methods rely on certain functional parameters. According to internationally recognized diagnostic criteria, the primary functional indicators for diagnosing CMD are:

Coronary Flow Reserve (CFR): This is the ratio of coronary blood flow in a maximally dilated state to the baseline coronary blood flow. It comprehensively reflects the potential blood supply capacity of both the epicardial coronary arteries and the coronary microcirculation. Drugs such as adenosine, dipyridamole, acetylcholine, regadenoson, and nicorandil can be used to achieve maximal dilation of the coronary arteries. The normal value of CFR is 3–5, and clinically, a CFR of less than 2.0 is recommended as the threshold for identifying microvascular dysfunction.

Index of Microcirculatory Resistance (IMR): Defined as the ratio of pressure (Pd) at the distal end of a stenotic lesion to 1/T under coronary hyperemic conditions, where pressure (Pd) and flow time (T) can be measured using a pressure wire equipped with a temperature sensor (82). IMR is independent of the function of epicardial vessels and can specifically assess the microvascular function at the distal end of a stenotic lesion with good reproducibility. An IMR of 25 or greater indicates microvascular dysfunction (82, 83).

Fractional Flow Reserve (FFR): This is the ratio of the maximum achievable blood flow to the myocardial region supplied by a stenotic artery to the theoretical maximum blood flow achievable under normal conditions in the same region. It is calculated as the ratio of the mean pressure (Pd) in the coronary artery distal to the stenosis to the mean aortic pressure (Pa) at the coronary orifice during maximal myocardial hyperemia. An FFR of 0.8 or less indicates myocardial ischemia, and an FFR of less than 0.75 suggests that the patient may benefit from revascularization.

Additionally, some indicators suggest the presence of CMD after PCI: (1) TIMI flow grade 0–2 post-PCI. (2) TIMI myocardial perfusion grade 0–2 post-PCI. (3) Less than 50% resolution of the ST-segment elevation at 90 min post-procedure. (4) Single-photon emission computed tomography (SPECT) showing areas of myocardial perfusion defects before discharge (84) (Table 2).

**Table 2:** Treatment methods for CMD after PCI.

Therapeutic Category	Treatment Strategy	Specific Drugs/Methods	Mechanism of Action/Therapeutic Goal (References)
Risk Factor Control	Hypertension Control	ACEIs, ARBs, Calcium Channel Blockers, β-Blockers	Improve microvascular perfusion, slow disease progression (96–98)
	Hyperlipidemia Control	Statins, PCSK9 Inhibitors (e.g., Evolocumab, Alirocumab)	Improve endothelial function, inhibit inflammatory response and oxidative stress, increase CFR (100–105)
	Diabetes Control	Metformin, Ticagrelor	Protect endothelial cells, improve insulin resistance; Antiplatelet, anti-inflammatory, and microvascular dilation effects (106–109)
Pre-PCI Prevention	Antiplatelet Therapy	Aspirin + Ticagrelor (or Clopidogrel)	Prevent microthrombus formation, reduce CMD occurrence (110)
	Statin Pretreatment	Statins	Improve coronary microvascular perfusion after revascularization in ACS patients (111)
	Chinese Medicine Pretreatment	Tongxinluo	Reduce intraoperative no-reflow phenomenon and myocardial infarction area (112)
Intra-PCI Intervention	Antiplatelet Drugs	Tirofiban, Abciximab, Eptifibatide	Reduce coronary microvascular obstruction, improve myocardial perfusion (113)
	Vasodilators	Adenosine, Nicorandil, Nitroprusside, Verapamil, Diltiazem	Improve coronary microcirculatory perfusion, reduce no-reflow (114–118)
	Non-Pharmacological Therapy	Thrombus Aspiration, Excimer Laser Ablation, Delayed Stent Implantation	For patients with high thrombus burden, reduce microvascular obstruction (119–121)
Post-PCI Treatment	Inhibit Inflammatory Response	Iron Chelators, β-Blockers	Reduce myocardial oxidative stress, inhibit neutrophil activation (123–125)
	Dilate Microvessels	Adenosine, Nitroprusside, Nicorandil, Atrial Natriuretic Peptide (ANP)	Relax vascular smooth muscle, reduce microvascular resistance, increase perfusion (126–129)
	Antiplatelet Therapy	Ticagrelor, Tirofiban	Inhibit platelet aggregation, prevent microthrombosis; Ticagrelor dilates microvessels via the adenosine pathway (6, 130)
	Non-Pharmacological Therapy	Ischemic Conditioning, Remote Ischemic Preconditioning	Induce the body's protective response against ischemia-reperfusion injury, reduce coronary microvascular obstruction, promote recovery (131–133)
Chinese Medicine Therapy	Integrated Chinese & Western Medicine	Shexiang Baoxin Pill, Qishen Yiqi Dripping Pill, Tongxinluo Capsule	Regulate inflammatory response and oxidative stress, reduce endothelial cell damage, improve microcirculatory function and quality of life through multiple mechanisms (134–140)

### 4.1 Invasive testing

Invasive testing mainly includes coronary angiography, bolus thermodilution method, continuous thermodilution method, and intracoronary Doppler flow velocity method.

Coronary angiography can be used to analyze the patency of the epicardial coronary arteries using the TIMI (Thrombolysis in Myocardial Infarction) flow grade and TIMI frame count methods, indirectly evaluating coronary microcirculation blood flow. However, the results are not precise and do not clearly identify the location or cause of the lesion (85). Currently, coronary microvascular function is often assessed through myocardial contrast enhancement speed, with specific indicators including the TIMI myocardial contrast enhancement grade, myocardial contrast density grade, and TIMI myocardial perfusion frame count method. TIMI myocardial contrast enhancement analysis and myocardial contrast density grading can classify coronary microcirculation into three levels, serving as semi-quantitative indicators for evaluating microcirculatory perfusion (86). The TIMI myocardial perfusion frame count method evaluates the patient's microcirculation based on the number of frames from myocardial contrast appearance to clearance, and studies have used this indicator to assess CMD after PCI (87).

The advantages of coronary angiography are that it allows immediate assessment of coronary microvascular function post-PCI and is straightforward to analyze. However, its limitations include the inability to measure CFR (coronary flow reserve), as the results are only semi-quantitative, and the analyzed indicators are easily influenced by heart rate and blood pressure.

Bolus Thermodilution Method involves injecting cold saline with a known temperature and injection rate into the coronary artery at its opening and measuring the degree of blood temperature drop. The extent of the temperature decrease reflects the microcirculatory perfusion, and the results are directly proportional to coronary blood flow (CBF) (88). Additionally, a temperature dilution curve can be constructed to calculate the mean transit time (T) of saline from the injection site to the sensor. By recording the ratio of T values under maximum and baseline conditions, CFR can be obtained. Its advantages include simplicity of operation and the ability to quantitatively analyze coronary microcirculatory blood flow. However, its disadvantages are that the manual operation of saline injection introduces variability, which can easily lead to overestimation of CFR values. Furthermore, the results are affected by factors such as heart rate, blood pressure, saline injection speed, and temperature, leading to some degree of variability in the measurement outcomes.

Continuous Thermodilution Method eliminates the influence of manual operation and saline injection speed on the test results by using a specialized monorail infusion catheter to inject room-temperature saline at a constant speed of 15–25 ml/min. A temperature-sensing wire first measures the blood temperature downstream and then measures the saline temperature at the distal end of the infusion catheter, allowing for the calculation of blood flow and resistance values. This method avoids the vascular stimulation caused by cold saline and reduces adverse reactions in patients (89, 90). Experiments have shown that the CFR values obtained by this method correlate well with those measured by PET (positron emission tomography) (91). However, the method still has limitations; an abnormal CFR detected by this method cannot distinguish whether the dysfunction is due to CMD or stenosis in the epicardial coronary arteries.

Intracoronary Doppler Flow Velocity Method records the coronary blood flow velocity at both baseline and hyperemic states. By calculating the ratio of flow velocities under these conditions, the CFR value is obtained. Additionally, this technique can use a pressure wire to measure the average blood flow velocity and pressure in the distal microvessels during maximal hyperemia, thereby determining the hyperemic microvascular resistance. Hyperemic microvascular resistance is calculated as the mean pressure divided by the mean velocity. When this value exceeds 1.7 mmHg/cm/s, and CFR is less than 2.5, CMD can be diagnosed (92). Studies have shown that hyperemic microvascular resistance is well correlated with clinical outcomes (93).

However, the drawback of this technique is its complexity in operation, and an abnormal CFR obtained does not definitively determine whether the cause is CMD or stenosis in the epicardial coronary arteries.

### 4.2 Non-Invasive testing

Non-invasive testing mainly includes techniques such as transthoracic coronary artery Doppler imaging, myocardial contrast echocardiography, single-photon emission computed tomography (SPECT), positron emission tomography (PET), cardiac magnetic resonance imaging (CMR), and computed tomography perfusion imaging (CTP).

Transthoracic color Doppler ultrasound, when using contrast agents, can visualize nearly 100% of the left anterior descending (LAD) artery and 54%–86% of the posterior descending artery (94). After fully dilating the coronary arteries using vasodilators, this method measures the peak diastolic flow velocity of the epicardial coronary arteries and compares it with the velocity at rest, obtaining the coronary flow velocity reserve (CFVR). CFVR has a high correlation with CFR and can generally be used interchangeably (95). A CFVR of ≤2.0 indicates the presence of coronary microvascular dysfunction (96).

The advantages of this method are that it is non-invasive, repeatable, highly feasible, has good patient compliance, is relatively inexpensive, and does not involve radiation exposure. However, the method's limitations include a high dependency on the operator's expertise and the precision of ultrasound imaging. Therefore, it is only suitable for clearly visualizing the LAD and its distal microvessels and is not applicable to branches, such as the circumflex artery, that cannot be clearly imaged (97).

Myocardial Contrast Echocardiography (MCE) primarily detects myocardial backscatter signals after intravenous injection of microbubble contrast agents. These backscatter signals can display myocardial blood flow (MBF) (98). By measuring MBF before and after the administration of vasodilator drugs, the myocardial CFR level of the patient can be obtained, directly quantifying the coronary microcirculation function. Clinical studies have shown that MBF measured by MCE is highly consistent with MBF obtained through PET (99). The advantages of MCE include the absence of radiation, simplicity of operation, and low cost. However, its quality is highly dependent on the operator's skill and can be easily affected by factors such as breathing and body position.

SPECT measures myocardial perfusion by recording the amount of radioactive tracers in the myocardium at rest and under stress, making it suitable for patients without epicardial coronary artery stenosis (100). SPECT/CT, when combined with low-dose CT scans, can co-localize perfusion with cardiac structures and correct for volume effects (101). The advantage of SPECT is its high sensitivity, making it an excellent negative predictive indicator. However, the limitations are that current routine SPECT cannot quantitatively measure CFR, and, given its radiation exposure, it has low spatial resolution, posing challenges for widespread clinical use.

PET calculates myocardial blood flow (MBF) per gram of myocardium per minute by detecting the radioactivity of radiotracer isotopes within the myocardium and constructing time-radioactivity curves for the left ventricular chamber and myocardium. By comparing MBF values before and after the administration of vasodilators, the coronary flow reserve (CFR) can be calculated. Currently, MBF and CFR measured by PET are considered the gold standard for diagnosing myocardial ischemia among non-invasive techniques (102, 103). Recently, combining PET with CT or MRI has overcome the attenuation effects of standalone PET imaging (104).

The advantages of PET are that it can accurately quantify MBF and CFR at rest and during stress, providing a precise assessment of myocardial perfusion. However, its disadvantages include high costs, long testing times, and radiation exposure. Additionally, when not combined with CT or MRI scans, PET has limited spatial resolution.

CMR enables simultaneous assessment of cardiac anatomy, myocardial function, and myocardial perfusion, while also providing a semi-quantitative myocardial perfusion reserve index (MPRI). A reduced MPRI indicates either the presence of CMD or increased resting myocardial perfusion (105). Recently, a study on a CMR respiratory motion correction myocardial perfusion measurement sequence demonstrated that MBF could be quantified, and CFR calculated while allowing the patient to breathe freely. The MBF values obtained by CMR are highly consistent with those measured by PET, confirming its diagnostic effectiveness (106). CMR can also assess myocardial perfusion using Dynamic Contrast-Enhanced Myocardial Perfusion Imaging. After intravenous injection of paramagnetic contrast agents, CMR can track the distribution and clearance process of contrast agents in the myocardium and blood vessels, and reflect the myocardial blood flow perfusion status through changes in signal intensity. Changes in signal intensity are used to reflect the state of myocardial blood perfusion. This method not only enables precise localization of perfusion defects by identifying areas with impaired perfusion but also calculates the extent of these defects through quantitative analysis, directly correlating with the coronary artery branches that are insufficiently supplying blood (107).

Furthermore, CMR can detect the presence of Microvascular Obstruction (MVO) in the coronary microvasculature. MVO leads to abnormal myocardial reperfusion in patients, affecting cardiac structure, function, and prognosis—manifestations include increased myocardial infarction size, reduced systolic function, poor recovery of systolic function, and heightened risk of adverse ventricular remodeling (108). Both early and late gadolinium enhancement sequences in CMR can visualize MVO. Since gadolinium-based contrast agents cannot pass through obstructed microvessels to enter myocardial tissue, MVO appears as a non-enhancing dark region on Late Gadolinium Enhancement (LGE) images. Notably, this dark region is located within the enhanced area of myocardial infarction, forming a characteristic pattern where the infarcted region surrounds the MVO. Studies have demonstrated that the presence or absence of early MVO following myocardial infarction in patients with STEMI is a crucial prognostic factor for revascularization outcomes in these patients (109).

The advantages of CMR are its high controllability, high spatial resolution, and ability to simultaneously measure function, morphology, and perfusion. This technique has gradually become the gold standard among non-invasive imaging techniques for diagnosing CMD (106, 110, 111). However, its drawbacks include the presence of artifacts in the subendocardial region, which can affect the calculation results. Additionally, the gadolinium-based contrast agents used in CMR have adverse effects on renal function, making it unsuitable for patients with renal impairment.

Computed Tomography Perfusion (CTP) is a myocardial functional imaging technique based on coronary computed tomography angiography (CTA). It is the only non-invasive technique that can simultaneously assess both the epicardial coronary artery and microcirculatory function (112). CTP has two scanning modes: the rest scan mode, which evaluates the stenosis of the epicardial coronary arteries, and the pharmacological stress scan mode, which allows for qualitative and quantitative evaluation of myocardial blood flow (113).

The capability of CTP to identify CMD is comparable to that of SPECT, and it can be performed alongside CTA, making it relatively low-cost with good patient compliance. This makes CTP particularly suitable for post-PCI patients. However, the drawbacks of CTP include higher radiation exposure and the inability to precisely quantify MBF and CFR. Additionally, there is no universally accepted diagnostic threshold, which limits its clinical application.

## 5 Treatment strategies

Firstly, since post-PCI CMD is often induced by the risk factors associated with atherosclerosis (AS), controlling the progression of diseases such as hypertension, hyperlipidemia, and diabetes is crucial for preventing and managing CMD. Secondly, preventive measures, including both pharmacological and non-pharmacological treatments, can be employed before and during PCI to address the causes of post-procedural CMD. Finally, for patients who develop CMD after PCI, a series of pharmacological and non-pharmacological treatments can be administered to improve their prognosis.

### 5.1 Risk factor control

Early and sustained control of blood pressure in patients with hypertension and CMD is crucial for slowing disease progression and improving patient prognosis. Studies have shown that antihypertensive drugs such as angiotensin-converting enzyme inhibitors (ACEIs), angiotensin receptor blockers (ARBs), calcium channel blockers, and β-blockers significantly improve microvascular perfusion (114–116). Trials have indicated that renal denervation therapy has a positive effect on patients with hypertension-related CMD, but previous research results have been controversial (116). In recent years, interventional procedures for treating microvascular angina have been under development, and the implantation of coronary sinus reducers has shown a positive impact on relieving angina symptoms in CMD patients by significantly reducing subendocardial vascular resistance (117).

For patients with hyperlipidemia and CMD, several small-scale studies have demonstrated that statins significantly increase exercise tolerance and CFR, improve exercise-induced poor tissue perfusion, and enhance the quality of life (118–121). The use of proprotein convertase subtilisin/kexin type 9 (PCSK9) inhibitors, such as evolocumab or alirocumab, not only lowers low-density lipoprotein cholesterol (LDL-C) but also significantly improves endothelial function, inhibits inflammatory responses, and reduces oxidative stress (122, 123).

Diabetic patients exhibit poorer post-PCI prognosis compared to non-diabetic patients (124), likely due to vascular endothelial damage induced by hyperglycemia, which promotes platelet adhesion, activation, and aggregation, ultimately leading to thrombus formation. Consequently, antiplatelet agents such as ticagrelor can alleviate myocardial ischemia symptoms in diabetic patients with CMD. In addition to its anti-inflammatory and antiplatelet functions, ticagrelor can also protect the myocardium from ischemia and reperfusion injury through its potent vasodilatory effects (125). A study on patients with ST-segment elevation acute coronary syndrome demonstrated that myocardial microcirculatory perfusion levels were significantly higher in diabetic patients treated with ticagrelor than with clopidogrel (126, 127). Furthermore, studies have shown that the antihyperglycemic agent metformin, in addition to reducing hepatic glucose output and improving insulin resistance, also exerts a protective effect on endothelial cells in diabetic patients, making it an ideal treatment for diabetes combined with CMD (128).

### 5.2 Prevention before and during PCI

Studies have shown that dual antiplatelet therapy before PCI can effectively prevent the occurrence of CMD. Currently, ticagrelor combined with aspirin is commonly used for antiplatelet therapy. For patients intolerant to ticagrelor or at high risk of bleeding, ticagrelor can be replaced with clopidogrel (129). Myocardial contrast echocardiography has shown that the use of statins before revascularization in patients with acute coronary syndrome can significantly improve coronary microvascular perfusion (6). In addition to Western medicine, taking a loading dose of the traditional Chinese medicine Tongxinluo before PCI in STEMI patients can significantly reduce the occurrence of intraoperative no-reflow and the myocardial infarction area 6 months post-procedure, although it does not significantly impact postoperative cardiovascular events (130).

During PCI, platelet glycoprotein IIb/IIIa receptor antagonists such as tirofiban, abciximab, or eptifibatide can be used to prevent post-PCI CMD. For PCI patients with a high thrombus burden, the use of platelet glycoprotein IIb/IIIa receptor antagonists either intracoronarily or intravenously can reduce the incidence of coronary microvascular obstruction, decrease myocardial infarction size, improve myocardial perfusion, and reduce rates of reinfarction and mortality (131). Additionally, multiple studies have demonstrated that using specific plasminogen activators, adenosine, nicorandil, nitroprusside, verapamil, and diltiazem during PCI can improve coronary microcirculatory perfusion and reduce the incidence of no-reflow post-procedure (132–136).

Non-pharmacological measures, such as thrombus aspiration, excimer laser ablation, and delayed stent implantation, can also be used to prevent post-PCI CMD. These non-pharmacological treatments are not routinely recommended, but for patients with a high thrombus burden, thrombus aspiration or excimer laser ablation can reduce coronary microvascular obstruction and improve myocardial microcirculation and perfusion (137, 138). Studies have shown that after thrombus aspiration or balloon dilation, achieving TIMI grade 3 flow and delaying stent implantation for 4 to 16 h can result in a lower incidence of no-reflow compared to direct PCI (139).

### 5.3 Post-PCI treatment

The ultimate goal of PCI is to reduce myocardial ischemia, protect cardiac function, and improve patient prognosis. Therefore, reopening the infarcted vessel is only the first step; subsequent treatment targeting CMD is of utmost importance. Post-PCI treatment mainly focuses on inhibiting inflammatory responses, dilating microvessels, and preventing platelet aggregation (140).

Due to tissue necrosis and ischemia-reperfusion injury, PCI patients often experience severe inflammatory responses. Experimental studies have demonstrated that several drugs can treat post-PCI inflammatory reactions. First, due to myocardial ischemia and intramyocardial hemorrhage, excessive iron deposition occurs in the myocardial interstitium. Research has found that administering iron chelators to post-PCI patients can significantly improve serum iron levels, reduce myocardial oxidative stress, and improve patient prognosis (141). Second, β-blockers can inhibit neutrophil activation (142). Studies have shown that, after β-blocker administration, patients with STEMI exhibit significantly improved left ventricular ejection fraction and microcirculatory perfusion, reduced myocardial infarct size, and notably better prognosis (143).

Excessive microvascular constriction is a crucial factor in the development of CMD, and treatments such as adenosine, nitroprusside, nicorandil, and atrial natriuretic peptide can be used. Adenosine relaxes vascular smooth muscle and helps to increase coronary microcirculatory perfusion. Studies have indicated that using adenosine during and after PCI can significantly reduce myocardial infarct size (144). Nicorandil, a nitrate drug and ATP-sensitive potassium channel opener, can dilate epicardial and coronary microvessels. A meta-analysis showed that perioperative use of nicorandil improves coronary blood flow, cardiac contractile function, and prognosis in STEMI patients undergoing initial PCI (145). Atrial natriuretic peptide (ANP) can reduce coronary microvascular obstruction by inhibiting endothelin-1 (146). Studies have found that administering ANP to STEMI patients before PCI significantly reduces myocardial infarct size (147).

Platelet aggregation is a major cause of microthrombus formation, and antiplatelet therapy is the first-line treatment for preventing and managing post-PCI CMD. Studies have shown that the antiplatelet drug ticagrelor not only inhibits platelet aggregation but also dilates microvessels via the adenosine pathway, thereby improving microcirculatory perfusion (148). Additionally, other studies have demonstrated that the use of the antiplatelet drug tirofiban can reduce microvascular damage and improve clinical outcomes (149).

Moreover, non-pharmacological treatments also play an important role in combating post-PCI CMD. Currently, clinical practice often employs ischemic conditioning to induce the body's protective response against ischemia-reperfusion injury after PCI. Ischemic conditioning involves repeatedly and briefly occluding the coronary artery with a balloon before PCI to cause transient myocardial ischemia, thereby reducing the occurrence of coronary microvascular obstruction and promoting left ventricular function recovery (150).

In addition, remote ischemic preconditioning involves using a cuff to repeatedly inflate and deflate the upper limb after PCI, inducing brief ischemia, which leads to the production of myocardial protective substances locally, thereby promoting myocardial survival (151). Research has shown that remote ischemic preconditioning can reduce myocardial infarct size, although it does not improve coronary blood flow. Follow-up studies have found that the group receiving remote ischemic preconditioning experienced significant reductions in all-cause mortality, cardiovascular events, and cerebrovascular events (152).

## 6 Summary and outlook

In summary, the occurrence of CMD after PCI significantly affects patient prognosis. Mechanistically, ischemia-reperfusion injury is the most critical factor contributing to post-PCI CMD. Reperfusion of ischemic myocardium induces severe oxidative stress, inflammatory responses, and endothelial dysfunction. Due to the small diameter of the vessels, current imaging techniques cannot directly observe the specific morphology of the coronary microcirculation and can only indirectly assess microcirculatory function through functional indicators. The commonly used diagnostic methods each have their advantages and disadvantages, but due to the limitations of these indirect indicators, these methods cannot effectively determine the exact location and cause of the lesions, let alone observe the progression of the disease.

Early detection and management of post-PCI CMD are crucial for improving patient outcomes. Therefore, identifying a simpler and more accessible method for post-PCI CMD detection is a key focus of future research in this field. Currently, the treatment strategies for post-PCI CMD mainly include risk factor control and pre-, intra-, and post-PCI interventions. However, these treatment strategies lack high-quality, large-scale, multicenter, randomized controlled clinical trials, and the specific therapeutic effects and molecular mechanisms are not yet fully understood.

Moreover, in clinical practice, numerous studies have demonstrated that traditional Chinese medicine (TCM) has good therapeutic and preventive effects on CMD after PCI. TCM can regulate post-PCI inflammatory responses, oxidative stress, and reduce endothelial cell damage through multiple mechanisms and pathways, thereby improving microcirculatory function, inflammation markers, and quality of life in post-PCI patients (153–158). Studies have shown that combining Chinese and Western medicine can significantly reduce the incidence of adverse cardiovascular events after PCI compared to Western medicine alone (159). However, due to the numerous components, pathways, and targets of TCM, exploring its specific active ingredients and mechanisms is challenging. Therefore, it is recommended to conduct more high-quality, large-sample, multicenter clinical studies while using advanced multi-omics analysis techniques to comprehensively and systematically study the specific mechanisms and effects of TCM.

## References

[1] Tsao CW Aday AW Almarzooq ZI Anderson CAM Arora P Avery CL Heart disease and stroke statistics-2023 update: a report from the American heart association. Circulation. (2023) 147(8):e93–e621. 10.1161/CIR.0000000000001123 36695182 PMC12135016

[2] Li X Liu S Liu H Zhu JJ. Acupuncture for gastrointestinal diseases. Anat Rec. (2023) 306(12):2997–3005. 10.1002/ar.24871 35148031

[3] Weferling M Hamm CW Kim WK. Percutaneous coronary intervention in transcatheter aortic valve implantation patients: overview and practical management. Front Cardiovasc Med. (2021) 8:653768. 10.3389/fcvm.2021.653768 34017866 PMC8129193

[4] De Luca L Rosano GMC Spoletini I. Post-percutaneous coronary intervention angina: from physiopathological mechanisms to individualized treatment. Cardiol J. (2022) 29(5):850–7. 10.5603/CJ.a2021.0042 33843042 PMC9550331

[5] Ajmal M Chatterjee A Acharya D. Persistent or recurrent angina following percutaneous coronary revascularization. Curr Cardiol Rep. (2022) 24(12):1837–48. 10.1007/s11886-022-01820-3 36287295

[6] Padro T Manfrini O Bugiardini R Canty J Cenko E De Luca G ESC Working group on coronary pathophysiology and microcirculation position paper on ‘coronary microvascular dysfunction in cardiovascular disease’. Cardiovasc Res. (2020) 116(4):741–55. 10.1093/cvr/cvaa003 32034397 PMC7825482

[7] Taqueti VR Di Carli MF. Coronary microvascular disease pathogenic mechanisms and therapeutic options: JACC state-of-the-art review. J Am Coll Cardiol. (2018) 72(21):2625–41. 10.1016/j.jacc.2018.09.042 30466521 PMC6296779

[8] Chinese Society of Cardiology, Chinese Medical Association; Editorial Board of Chinese Journal of Cardiology. Chinese Expert consensus on diagnosis and management on patients with ischemia and non-obstructive coronary artery disease. Zhonghua Xin Xue Guan Bing Za Zhi. (2022) 50(12):1148–60. 10.3760/cma.j.cn112148-20220908-00682 36517435

[9] Jansen TPJ de Vos A Paradies V Damman P Teerenstra S Konst RE Absolute flow and resistance have superior repeatability as compared to CFR and IMR: EDIT-CMD substudy. JACC Cardiovasc Interv. (2023) 16(7):872–4. 10.1016/j.jcin.2022.11.019 36898940

[10] Buono D Montone MG Camilli RA Carbone M Narula S Lavie J Coronary microvascular dysfunction across the spectrum of cardiovascular diseases: JACC state-of-the-art review. J Am Coll Cardiol. (2021) 78(13):1352–71. 10.1016/j.jacc.2021.07.042 34556322 PMC8528638

[11] Vancheri F Longo G Vancheri S Henein M. Coronary microvascular dysfunction. J Clin Med. (2020) 9(9):2880. 10.3390/jcm9092880 32899944 PMC7563453

[12] Bhatt DL Lopes RD Harrington RA. Diagnosis and treatment of acute coronary syndromes: a review. JAMA. (2022) 327(7):662–75. 10.1001/jama.2022.0358 35166796

[13] Salvatore T Galiero R Caturano A Vetrano E Loffredo G Rinaldi L Coronary microvascular dysfunction in diabetes mellitus: pathogenetic mechanisms and potential therapeutic options. Biomedicines. (2022) 10(9):2274. 10.3390/biomedicines10092274 36140374 PMC9496134

[14] Chandurkar MK Mittal N Royer-Weeden SP Lehmann SD Rho Y Han SJ. Low Shear in Short-Term Impacts Endothelial Cell Traction and Alignment in Long-Term. bioRxiv: the preprint server for biology. (2024). 10.1152/ajpheart.00067.2024 PMC11649189 38457352

[15] Zhang L Li Y Ma X Liu J Wang X Zhang L Ginsenoside Rg1-notoginsenoside R1-protocatechuic aldehyde reduces atherosclerosis and attenuates low-shear stress-induced vascular endothelial cell dysfunction. Front Pharmacol. (2020) 11:588259. 10.3389/fphar.2020.588259 33568993 PMC7868340

[16] Hauger PC Hordijk PL. Shear stress-induced AMP-activated protein kinase modulation in endothelial cells: its role in metabolic adaptions and cardiovascular disease. Int J Mol Sci. (2024) 25(11):6047. 10.3390/ijms25116047 38892235 PMC11173107

[17] Bibli SI Hu J Looso M Weigert A Ratiu C Wittig J Mapping the endothelial cell S-sulfhydrome highlights the crucial role of integrin sulfhydration in vascular function. Circulation. (2021) 143(9):935–48. 10.1161/CIRCULATIONAHA.120.051877 33307764

[18] Pu L Meng Q Li S Wang Y Liu B. TXNRD1 Knockdown inhibits the proliferation of endothelial cells subjected to oscillatory shear stress via activation of the endothelial nitric oxide synthase/apoptosis pathway. Biochim Biophys Acta Mol Cell Res. (2023) 1870(4):119436. 10.1016/j.bbamcr.2023.119436 36754152

[19] Wei W Tang M Wang Q Li X. Circ_HECW2 regulates ox-LDL-induced dysfunction of cardiovascular endothelial cells by miR-942-5p/TLR4 axis. Clin Hemorheol Microcir. (2025) 89(1):1–14. 10.3233/CH-221550 36213989

[20] Ma FX Zhou B Chen Z Ren Q Lu SH Sawamura T Oxidized low density lipoprotein impairs endothelial progenitor cells by regulation of endothelial nitric oxide synthase. J Lipid Res. (2006) 47(6):1227–37. 10.1194/jlr.M500507-JLR200 16522925

[21] Cheng H Zhong W Wang L Zhang Q Ma X Wang Y Effects of shear stress on vascular endothelial functions in atherosclerosis and potential therapeutic approaches. Biomed Pharmacother. (2023) 158:114198. 10.1016/j.biopha.2022.114198 36916427

[22] Ji Q Wang YL Xia LM Yang Y Wang CS Mei YQ. High shear stress suppresses proliferation and migration but promotes apoptosis of endothelial cells co-cultured with vascular smooth muscle cells via down-regulating MAPK pathway. J Cardiothorac Surg. (2019) 14(1):216. 10.1186/s13019-019-1025-5 31831023 PMC6909635

[23] Dubsky M Veleba J Sojakova D Marhefkova N Fejfarova V Jude EB. Endothelial dysfunction in diabetes mellitus: new insights. Int J Mol Sci. (2023) 24(13):10705. 10.3390/ijms241310705 37445881 PMC10341633

[24] Sun D Wang J Toan S Muid D Li R Chang X Molecular mechanisms of coronary microvascular endothelial dysfunction in diabetes mellitus: focus on mitochondrial quality surveillance. Angiogenesis. (2022) 25(3):307–29. 10.1007/s10456-022-09835-8 35303170

[25] Scioli MG Storti G D’Amico F Rodríguez Guzmán R Centofanti F Doldo E Oxidative stress and new pathogenetic mechanisms in endothelial dysfunction: potential diagnostic biomarkers and therapeutic targets. J Clin Med. (2020) 9(6):1995. 10.3390/jcm9061995 32630452 PMC7355625

[26] Yang X-X Zhao Z-Y. Pharmacology. miR-30a-5p inhibits the proliferation and collagen formation of cardiac fibroblasts in diabetic cardiomyopathy. Can J Physiol Pharmacol. (2022) 100(2):167–75. 10.1139/cjpp-2021-0280 35025607

[27] Li J Salvador AM Li G Valkov N Ziegler O Yeri A Mir-30d regulates cardiac remodeling by intracellular and paracrine signaling. Circ Res. (2021) 128(1):e1–e23. 10.1161/CIRCRESAHA.120.317244 33092465 PMC7790887

[28] Bonnardel-Phu E Wautier J-L Schmidt AM Avila C Vicaut EJD. Acute modulation of albumin microvascular leakage by advanced glycation end products in microcirculation of diabetic rats *in vivo*. Diabetes. (1999) 48(10):2052–8. 10.2337/diabetes.48.10.2052 10512373

[29] Gupta JK. The role of aldose reductase in polyol pathway: an emerging pharmacological target in diabetic complications and associated morbidities. Curr Pharm Biotechnol. (2024) 25(9):1073–81. 10.2174/1389201025666230830125147 37649296

[30] Adamska A Araszkiewicz A Pilacinski S Gandecka A Grzelka A Kowalska K Dermal microvessel density and maturity is closely associated with atherogenic dyslipidemia and accumulation of advanced glycation end products in adult patients with type 1 diabetes. Microvasc Res. (2019) 121:46–51. 10.1016/j.mvr.2018.10.002 30312628

[31] Dong H Zhang Y Huang Y Deng H. Pathophysiology of RAGE in inflammatory diseases. Front Immunol. (2022) 13:931473. 10.3389/fimmu.2022.931473 35967420 PMC9373849

[32] Wang B Jiang T Qi Y Luo S Xia Y Lang B AGE-RAGE axis and cardiovascular diseases: pathophysiologic mechanisms and prospects for clinical applications. Cardiovasc Drugs Ther. (2024) 2024(Nov 5):1–18. 10.1007/s10557-024-07639-0 PMC12717123 39499399

[33] Jaap AJ Tooke JE. Diabetes and the Microcirculation. Clinically Applied Microcirculation Research. London: Routledge (2019). p. 31–44.

[34] Vinik AI Erbas T Casellini CM. Diabetic cardiac autonomic neuropathy, inflammation and cardiovascular disease. J Diabetes Investig. (2013) 4(1):4–18. 10.1111/jdi.12042 23550085 PMC3580884

[35] Feihl F Liaudet L Levy BI Waeber B. Hypertension and microvascular remodelling. Cardiovasc Res. (2008) 78(2):274–85. 10.1093/cvr/cvn022 18250145

[36] Zeng X Yang Y. Molecular mechanisms underlying vascular remodeling in hypertension. Rev Cardiovasc Med. (2024) 25(2):72. 10.31083/j.rcm2502072 39077331 PMC11263180

[37] Chung Y. Oxygen reperfusion is limited in the postischemic hypertrophic myocardium. Am J Physiol Heart Circ Physiol. (2006) 290(5):H2075–H84. 10.1152/ajpheart.00619.200 16603707

[38] Goumans M-J Ten Dijke P. TGF-β signaling in control of cardiovascular function. Cold Spring Harb Perspect Biol. (2018) 10(2):a022210. 10.1101/cshperspect.a022210 28348036 PMC5793760

[39] Grassi G Mark A Esler M. The sympathetic nervous system alterations in human hypertension. Circ Res. (2015) 116(6):976–90. 10.1161/CIRCRESAHA.116.303604 25767284 PMC4367954

[40] Hermann M Flammer A Lüscher TF. Nitric oxide in hypertension. J Clin Hypertens. (2006) 8:17–29. 10.1111/j.1524-6175.2006.06032.x PMC8109558 17170603

[41] Ajoolabady A Pratico D Ren J. Angiotensin II: role in oxidative stress, endothelial dysfunction, and diseases. Mol Cell Endocrinol. (2024) 592:112309. 10.1016/j.mce.2024.112309 38852657

[42] Zhan B Xu Z Zhang Y Wan K Deng H Wang D Nicorandil reversed homocysteine-induced coronary microvascular dysfunction via regulating PI3K/Akt/eNOS pathway. Biomed Pharmacother. (2020) 127:110121. 10.1016/j.biopha.2020.11012 32407984

[43] Durante A Mazzapicchi A Baiardo Redaelli M. Systemic and cardiac microvascular dysfunction in hypertension. Int J Mol Sci. (2024) 25(24):13294. 10.3390/ijms252413294 39769057 PMC11677602

[44] Puranik AS Dawson ER Peppas NA. Recent advances in drug eluting stents. Int J Pharm. (2013) 441(1–2):665–79. 10.1016/j.ijpharm.2012.10.029 23117022 PMC3567608

[45] van Beusekom HM Whelan DM Hofma SH Krabbendam SC van Hinsbergh VW Verdouw PD Long-term endothelial dysfunction is more pronounced after stenting than after balloon angioplasty in porcine coronary arteries. J Am Coll Cardiol. (1998) 32(4):1109–17. 10.1016/S0735-1097(98)00348-9 9768740

[46] van der Giessen WJ Sorop O Serruys PW Peters-Krabbendam I van Beusekom HM. Lowering the dose of sirolimus, released from a nonpolymeric hydroxyapatite coated coronary stent, reduces signs of delayed healing. JACC Cardiovasc Interv. (2009) 2(4):284–90. 10.1016/j.jcin.2008.12.012 19463438

[47] Klugherz BD Llanos G Lieuallen W Kopia GA Papandreou G Narayan P Twenty-eight-day efficacy and phamacokinetics of the sirolimus-eluting stent. Coron Artery Dis. (2002) 13(3):183–8. 10.1097/00019501-200205000-00008 12131023

[48] Finn AV Kolodgie FD Harnek J Guerrero LJ Acampado E Tefera K Differential response of delayed healing and persistent inflammation at sites of overlapping sirolimus- or paclitaxel-eluting stents. Circulation. (2005) 112(2):270–8. 10.1161/CIRCULATIONAHA.104.508937 15998681

[49] Li Y Yang D Lu L Wu D Yao J Hu X Thermodilutional confirmation of coronary microvascular dysfunction in patients with recurrent angina after successful percutaneous coronary intervention. Can J Cardiol. (2015) 31(8):989–97. 10.1016/j.cjca.2015.03.004 26088108

[50] Godo S Suda A Takahashi J Yasuda S Shimokawa H. Coronary microvascular dysfunction. Arterioscler, Thromb, Vasc Biol. (2021) 41(5):1625–37. 10.1161/ATVBAHA.121.316025 33761763

[51] Jabs A Münzel TJ. Drug-eluting stent-induced vascular dysfunction. J Am Coll Cardiol. (2010) 55(13):1399. 10.1016/j.jacc.2009.12.024 20338508

[52] Fedele G Castiglioni S Maier JA Locatelli L. The effects of sirolimus and magnesium on primary human coronary endothelial cells: an *in vitro* study. Int J Mol Sci. (2023) 24(3):2930. 10.3390/ijms24032930 36769252 PMC9917770

[53] Nebeker JR Virmani R Bennett CL Hoffman JM Samore MH Alvarez J Hypersensitivity cases associated with drug-eluting coronary stents: a review of available cases from the research on adverse drug events and reports (RADAR) project. J Am Coll Cardiol. (2006) 47(1):175–81. 10.1016/j.jacc.2005.07.071 16386683

[54] Patschan D Witzke O Dührsen U Erbel R Philipp T Herget-Rosenthal SJ. Acute myocardial infarction in thrombotic microangiopathies—clinical characteristics, risk factors and outcome. Nephrol Dial Transplant. (2006) 21(6):1549–54. 10.1093/ndt/gfl127 16574680

[55] van Lavieren MA van de Hoef TP Piek JJ. Primary PCI: time to change focus from epicardial reperfusion towards protection of the microvasculature. EuroIntervention. (2014) 115:T39–46. 10.4244/EIJV10STA8 25256533

[56] Herem JW. Mural platelet microthrombi and major acute lesions of main epicardial arteries in sudden coronary death. Atherosclerosis. (1974) 19(3):529–41. 10.1016/s0021-9150(74)80017-1 4828572

[57] Jaffe R Dick A Strauss BH. Prevention and treatment of microvascular obstruction-related myocardial injury and coronary no-reflow following percutaneous coronary intervention: a systematic approach. JACC Cardiovasc Interv. (2010) 3(7):695–704. 10.1016/j.jcin.2010.05.004 20650430

[58] Brandes RP Weissmann N Schröder K. Nox family NADPH oxidases: molecular mechanisms of activation. Free Radical Biol Med. (2014) 76:208–26. 10.1016/j.freeradbiomed.2014.07.046 25157786

[59] Faro DC Di Pino FL Monte IP. Inflammation, oxidative stress, and endothelial dysfunction in the pathogenesis of vascular damage: unraveling novel cardiovascular risk factors in fabry disease. Int J Mol Sci. (2024) 25(15):8273. 10.3390/ijms25158273 39125842 PMC11312754

[60] Crea F Lanza GA Camici PG Crea F Lanza GA Camici PG. CMD In the absence of myocardial diseases and obstructive CAD. In: Coronary Microvascular Dysfunction. Milano: Springer (2014). p. 75–114. 10.1007/978-88-470-5367-0_4

[61] Zhang Z Li X He J Wang S Wang J Liu J Molecular mechanisms of endothelial dysfunction in coronary microcirculation dysfunction. J Thromb Thrombolysis. (2023) 56(3):388–97. 10.1007/s11239-023-02862-2 37466848

[62] Panieri E Santoro MM. ROS Signaling and redox biology in endothelial cells. Cellular and molecular life sciences: CMLS. Cell Mol Life Sci. (2015) 72(17):3281–303. 10.1007/s00018-015-1928-9 25972278 PMC11113497

[63] Magenta A Greco S Capogrossi MC Gaetano C Martelli F. Nitric oxide, oxidative stress, and p66Shc interplay in diabetic endothelial dysfunction. BioMed Res Int. (2014) 2014:193095. 10.1155/2014/193095 24734227 PMC3964753

[64] Godo S Takahashi J Yasuda S Shimokawa H. Endothelium in coronary macrovascular and microvascular diseases. J Cardiovasc Pharmacol. (2021) 78(6):S19–s29. 10.1097/FJC.0000000000001089 34840261 PMC8647695

[65] He X Zhao M Bi X-Y Yu X-J Zang W-J. Delayed preconditioning prevents ischemia/reperfusion-induced endothelial injury in rats: role of ROS and eNOS. Lab Invest. (2013) 93(2):168–80. 10.1038/labinvest.2012.160 23147223

[66] Liu P Hock CE Nagele R Wong PY. Formation of nitric oxide, superoxide, and peroxynitrite in myocardial ischemia-reperfusion injury in rats. Am J Physiol. (1997) 272(5):H2327–H36. 10.1152/ajpheart.1997.272.5.h2327 9176302

[67] Piacenza L Zeida A Trujillo M Radi R. The superoxide radical switch in the biology of nitric oxide and peroxynitrite. Physiol Rev. (2022) 102(4):1881–906. 10.1152/physrev.00005.2022 35605280

[68] Gantzer J. eNOS and BH4; endothelial function or dysfunction. Importance of tetrahydrobiopterin (BH4). J Neurol Clin Neurosc. (2018) 2(3):1. 10.13140/RG.2.2.26142.18246

[69] Takata T Araki S Tsuchiya Y Watanabe Y. Oxidative stress orchestrates MAPK and nitric-oxide synthase signal. Int J Mol Sci. (2020) 21(22):8750. 10.3390/ijms21228750 33228180 PMC7699490

[70] Karbach S Wenzel P Waisman A Munzel T Daiber A. eNOS uncoupling in cardiovascular diseases-the role of oxidative stress and inflammation. Curr Pharm Des. (2014) 20(22):3579–94. 10.2174/13816128113196660748 24180381

[71] Daiber A Di Lisa F Oelze M Kröller-Schön S Steven S Schulz E Crosstalk of mitochondria with NADPH oxidase via reactive oxygen and nitrogen species signalling and its role for vascular function. Br J Pharmacol. (2017) 174(12):1670–89. 10.1111/bph.13403 26660451 PMC5446573

[72] Zhao J Li J Li G Chen M. The role of mitochondria-associated membranes mediated ROS on NLRP3 inflammasome in cardiovascular diseases. Front Cardiovasc Med. (2022) 9:1059576. 10.3389/fcvm.2022.1059576 36588561 PMC9794868

[73] Abais JM Xia M Zhang Y Boini KM Li P-L. Redox regulation of NLRP3 inflammasomes: rOS as trigger or effector? Antioxid Redox Signal. (2015) 22(13):1111–29. 10.1089/ars.2014.5994 25330206 PMC4403231

[74] He Y Hara H Núñez G. Mechanism and regulation of NLRP3 inflammasome activation. Trends Biochem Sci. (2016) 41(12):1012–21. 10.1016/j.tibs.2016.09.002 27669650 PMC5123939

[75] Kim S-K Park K-Y Choe J-Y. Toll-like receptor 9 is involved in NLRP3 inflammasome activation and IL-1β production through monosodium urate-induced mitochondrial DNA. Inflammation. (2020) 43(6):2301–11. 10.1007/s10753-020-01299-6 32700178

[76] Lee B. Understanding the Mechanisms That Drive NLRP3-Dependent Inflammation. United Kingdom: The University of Manchester (2022).

[77] Tang F Awad MA. Calpain-1 mediated mitochondria ROS/NLRP3 inflammasome in atherosclerosis. J Biosci Med. (2023) 11(4):50–9. 10.4236/jbm.2023.114005

[78] Ravindran J Gupta N Agrawal M Bala Bhaskar A Lakshmana Rao P. Modulation of ROS/MAPK signaling pathways by okadaic acid leads to cell death via, mitochondrial mediated caspase-dependent mechanism. Apoptosis. (2011) 16:145–61. 10.1007/s10495-010-0554-0 21082355

[79] Penninger JM Kroemer G. Mitochondria, AIF and caspases—rivaling for cell death execution. Nat Cell Biol. (2003) 5(2):97–9. 10.1038/ncb0203-97 12563272

[80] Kim J-Y Park J-H. ROS-dependent caspase-9 activation in hypoxic cell death. FEBS Lett. (2003) 549(1–3):94–8. 10.1016/S0014-5793(03)00795-6 12914932

[81] Badiger RH Dinesha V Hosalli A Ashwin SP. hs-C-reactive protein as an indicator for prognosis in acute myocardial infarction. J Sci Soc. (2014) 41(2):118–21. 10.4103/0974-5009.132859

[82] Fearon WF Balsam LB Farouque HO Robbins RC Fitzgerald PJ Yock PG Novel index for invasively assessing the coronary microcirculation. Circulation. (2003) 107(25):3129–32. 10.1161/01.CIR.0000080700.98607.D1 12821539

[83] Mangiacapra F Peace AJ Di Serafino L Pyxaras SA Bartunek J Wyffels E Intracoronary EnalaPrilat to reduce MICROvascular damage during percutaneous coronary intervention (ProMicro) study. J Am Coll Cardiol. (2013) 61(6):615–21. 10.1016/j.jacc.2012.11.025 23290547

[84] Chen W Ni M Huang H Cong H Fu X Gao W Chinese Expert consensus on the diagnosis and treatment of coronary microvascular diseases (2023 edition). MedComm. (2023) 4(6):e438. 10.1002/mco2.438 38116064 PMC10729292

[85] Gibson CM Cannon CP Daley WL Dodge JT Jr. Alexander B Jr. Marble SJ TIMI Frame count: a quantitative method of assessing coronary artery flow. Circulation. (1996) 93(5):879–88. 10.1161/01.CIR.93.5.879 8598078

[86] Molloi S Ersahin A Tang J Hicks J Leung CY. Quantification of volumetric coronary blood flow with dual-energy digital subtraction angiography. Circulation. (1996) 93(10):1919–27. 10.1161/01.CIR.93.10.1919 8635272

[87] Ge H Ding S An D Li Z Ding H Yang F Frame counting improves the assessment of post-reperfusion microvascular patency by TIMI myocardial perfusion grade: evidence from cardiac magnetic resonance imaging. Int J Cardiol. (2016) 203:360–6. 10.1016/j.ijcard.2015.10.194 26539957

[88] Pijls NH De Bruyne B Smith L Aarnoudse W Barbato E Bartunek J Coronary thermodilution to assess flow reserve: validation in humans. Circulation. (2002) 105(21):2482–6. 10.1161/01.CIR.0000017199.09457.3D 12034653

[89] Adjedj J Picard F Collet C Bruneval P Fournier S Bize A Intracoronary saline-induced hyperemia during coronary thermodilution measurements of absolute coronary blood flow: an animal mechanistic study. J Am Heart Assoc. (2020) 9(15):e015793. 10.1161/JAHA.120.015793 32689859 PMC7792254

[90] Jansen TPJ Konst RE Elias-Smale SE van den Oord SC Ong P de Vos AMJ Assessing microvascular dysfunction in angina with unobstructed coronary arteries: JACC review topic of the week. J Am Coll Cardiol. (2021) 78(14):1471–9. 10.1016/j.jacc.2021.08.028 34593129

[91] Everaars H de Waard GA Schumacher SP Zimmermann FM Bom MJ van de Ven PM Continuous thermodilution to assess absolute flow and microvascular resistance: validation in humans using [15O]H2O positron emission tomography. Eur Heart J. (2019) 40(28):2350–9. 10.1093/eurheartj/ehz245 31327012

[92] Xaplanteris P Fournier S Keulards DCJ Adjedj J Ciccarelli G Milkas A Catheter-based measurements of absolute coronary blood flow and microvascular resistance: feasibility, safety, and reproducibility in humans. Circ Cardiovasc Interv. (2018) 11(3):e006194. 10.1161/CIRCINTERVENTIONS.117.006194 29870386

[93] de Waard GA Fahrni G de Wit D Kitabata H Williams R Patel N Hyperaemic microvascular resistance predicts clinical outcome and microvascular injury after myocardial infarction. Heart. (2018) 104(2):127–34. 10.1136/heartjnl-2017-311431 28663361

[94] Lethen H PT H Kersting S Lambertz H. Validation of noninvasive assessment of coronary flow velocity reserve in the right coronary artery. A comparison of transthoracic echocardiographic results with intracoronary Doppler flow wire measurements. Eur Heart J. (2003) 24(17):1567–75. 10.1016/S0195-668X(03)00284-7 12927192

[95] Vegsundvåg J Holte E Wiseth R Hegbom K Hole T. Coronary flow velocity reserve in the three main coronary arteries assessed with transthoracic Doppler: a comparative study with quantitative coronary angiography. J Am Soc Echocardiogr. (2011) 24(7):758–67. 10.1016/j.echo.2011.03.010 21524564

[96] Ong P Camici PG Beltrame JF Crea F Shimokawa H Sechtem U International standardization of diagnostic criteria for microvascular angina. Int J Cardiol. (2018) 250:16–20. 10.1016/j.ijcard.2017.08.068 29031990

[97] Ong P Safdar B Seitz A Hubert A Beltrame JF Prescott E. Diagnosis of coronary microvascular dysfunction in the clinic. Cardiovasc Res. (2020) 116(4):841–55. 10.1093/cvr/cvz339 31904824

[98] Wei K Jayaweera AR Firoozan S Linka A Skyba DM Kaul S. Quantification of myocardial blood flow with ultrasound-induced destruction of microbubbles administered as a constant venous infusion. Circulation. (1998) 97(5):473–83. 10.1161/01.CIR.97.5.473 9490243

[99] Vogel R Indermühle A Reinhardt J Meier P Siegrist PT Namdar M The quantification of absolute myocardial perfusion in humans by contrast echocardiography: algorithm and validation. J Am Coll Cardiol. (2005) 45(5):754–62. 10.1016/j.jacc.2004.11.044 15734622

[100] Tona F Montisci R Iop L Civieri G. Role of coronary microvascular dysfunction in heart failure with preserved ejection fraction. Rev Cardiovasc Med. (2021) 22(1):97–104. 10.31083/j.rcm.2021.01.277 33792251

[101] Zavadovsky KV Mochula AV Maltseva AN Shipulin VV Sazonova SI Gulya MO The current status of CZT SPECT myocardial blood flow and reserve assessment: tips and tricks. J Nucl Cardiol. (2022) 29(6):3137–51. 10.1007/s12350-021-02620-y 33939162

[102] Brainin P Frestad D Prescott E. The prognostic value of coronary endothelial and microvascular dysfunction in subjects with normal or non-obstructive coronary artery disease: a systematic review and meta-analysis. Int J Cardiol. (2018) 254:1–9. 10.1016/j.ijcard.2017.10.052 29407076

[103] Klein R Ocneanu A Renaud JM Ziadi MC Beanlands RSB deKemp RA. Consistent tracer administration profile improves test-retest repeatability of myocardial blood flow quantification with (82)Rb dynamic PET imaging. J Nucl Cardiol. (2018) 25(3):929–41. 10.1007/s12350-016-0698-6 27804067 PMC5966478

[104] Fonti R Conson M Del Vecchio S. PET/CT in radiation oncology. Semin Oncol. (2019) 46(3):202–9. 10.1053/j.seminoncol.2019.07.001 31378377

[105] Bella D Parker EV Sinusas DL J A. On the dark rim artifact in dynamic contrast-enhanced MRI myocardial perfusion studies. Magn Reson Med. (2005) 54(5):1295–9. 10.1002/mrm.20666 16200553 PMC2377407

[106] Kotecha T Martinez-Naharro A Boldrini M Knight D Hawkins P Kalra S Automated pixel-wise quantitative myocardial perfusion mapping by CMR to detect obstructive coronary artery disease and coronary microvascular dysfunction: validation against invasive coronary physiology. JACC Cardiovasc Imaging. (2019) 12(10):1958–69. 10.1016/j.jcmg.2018.12.022 30772231 PMC8414332

[107] Li X-M Jiang L Min C-Y Yan W-F Shen M-T Liu X-J Myocardial perfusion imaging by cardiovascular magnetic resonance: research progress and current implementation. Curr Probl Cardiol. (2023) 48(6):101665. 10.1016/j.cpcardiol.2023.101665 36828047

[108] Nicolau AM Silva PG Mejía HPG Granada JF Kaluza GL Burkhoff D Molecular mechanisms of microvascular obstruction and dysfunction in percutaneous coronary interventions: from pathophysiology to therapeutics—a comprehensive review. Int J Mol Sci. (2025) 26(14):6835. 10.3390/ijms26146835 40725082 PMC12295318

[109] Kellman PJJCI. Dark-blood Late-enhancement imaging Improves Detection of Myocardial Infarction. Washington, DC: American College of Cardiology Foundation (2018). p. 1770–2. 10.1016/j.jcmg.2017.10.014 29248642

[110] Engblom H Xue H Akil S Carlsson M Hindorf C Oddstig J Fully quantitative cardiovascular magnetic resonance myocardial perfusion ready for clinical use: a comparison between cardiovascular magnetic resonance imaging and positron emission tomography. J Cardiovasc Magn Reson. (2017) 19(1):78. 10.1186/s12968-017-0388-9 29047385 PMC5648469

[111] Manka R Wissmann L Gebker R Jogiya R Motwani M Frick M Multicenter evaluation of dynamic three-dimensional magnetic resonance myocardial perfusion imaging for the detection of coronary artery disease defined by fractional flow reserve. Circ Cardiovasc Imaging. (2015) 8(5):e003061. 10.1161/CIRCIMAGING.114.003061 25901043

[112] Rossi A Merkus D Klotz E Mollet N de Feyter PJ Krestin GP. Stress myocardial perfusion: imaging with multidetector CT. Radiology. (2014) 270(1):25–46. 10.1148/radiol.13112739 24354374

[113] Danad I Szymonifka J Schulman-Marcus J Min JK. Static and dynamic assessment of myocardial perfusion by computed tomography. Eur Heart J Cardiovasc Imaging. (2016) 17(8):836–44. 10.1093/ehjci/jew044 27013250 PMC4955293

[114] Ong P Athanasiadis A Sechtem U. Pharmacotherapy for coronary microvascular dysfunction. Eur Heart J Cardiovasc Pharmacother. (2015) 1(1):65–71. 10.1093/ehjcvp/pvu020 27533969

[115] Michelsen MM Rask AB Suhrs E Raft KF Høst N Prescott E. Effect of ACE-inhibition on coronary microvascular function and symptoms in normotensive women with microvascular angina: a randomized placebo-controlled trial. PLoS One. (2018) 13(6):e0196962. 10.1371/journal.pone.0196962 29883497 PMC5993253

[116] Soleymani M Masoudkabir F Shabani M Vasheghani-Farahani A Behnoush AH Khalaji AJCT. Updates on pharmacologic management of microvascular angina. Cardiovasc Ther. (2022) 2022(1):6080258. 10.1155/2022/6080258 36382021 PMC9626221

[117] Ullrich H Hammer P Olschewski M Münzel T Escaned J Gori T. Coronary venous pressure and microvascular hemodynamics in patients with microvascular angina: a randomized clinical trial. JAMA Cardiol. (2023) 8(10):979–83. 10.1001/jamacardio.2023.2566 37610757 PMC10448373

[118] Yong J Tian J Yang X Xing H He Y Song X. Effects of oral drugs on coronary microvascular function in patients without significant stenosis of epicardial coronary arteries: a systematic review and meta-analysis of coronary flow reserve. Front Cardiovasc Med. (2020) 7:580419. 10.3389/fcvm.2020.580419 33195465 PMC7661556

[119] Ford T Berry C. How to diagnose and manage angina without obstructive coronary artery disease: lessons from the British heart foundation CorMicA trial. Interv Cardiol Revc. (2019) 14(2):76. 10.15420/icr.2019.04.R1 PMC6545998 31178933

[120] Ridker PM MacFadyen J Libby P Glynn RJ. Relation of baseline high-sensitivity C-reactive protein level to cardiovascular outcomes with rosuvastatin in the justification for use of statins in prevention: an intervention trial evaluating rosuvastatin (JUPITER). Am J Cardiol. (2010) 106(2):204–9. 10.1016/j.amjcard.2010.03.018 20599004

[121] Zhang X Li Q Zhao J Li X Sun X Yang H Effects of combination of statin and calcium channel blocker in patients with cardiac syndrome X. Coron Artery Dis. (2014) 25(1):40–4. 10.1097/MCA.0000000000000054 24256699

[122] Schremmer J Busch L Baasen S Heinen Y Sansone R Heiss C Chronic PCSK9 inhibitor therapy leads to sustained improvements in endothelial function, arterial stiffness, and microvascular function. Microvasc Res. (2023) 148:104513. 10.1016/j.mvr.2023.104513 36870561

[123] Nicholls SJ Puri R Anderson T Ballantyne CM Cho L Kastelein JJ Effect of evolocumab on progression of coronary disease in statin-treated patients: the GLAGOV randomized clinical trial. JAMA. (2016) 316(22):2373–84. 10.1001/jama.2016.16951 27846344

[124] Moses JW Mehran R Dangas GD Kobayashi Y Lansky AJ Mintz GS Short-and long-term results after multivessel stenting in diabetic patients. J Am Coll Cardiol. (2004) 43(8):1348–54. 10.1016/j.jacc.2003.04.004 15093865

[125] Cattaneo M Schulz R Nylander S. Adenosine-mediated effects of ticagrelor: evidence and potential clinical relevance. J Am Coll Cardiol. (2014) 63(23):2503–9. 10.1016/j.jacc.2014.03.031 24768873

[126] Echavarría-Pinto M Gonzalo N Ibañez B Petraco R Jimenez-Quevedo P Sen S Low coronary microcirculatory resistance associated with profound hypotension during intravenous adenosine infusion: implications for the functional assessment of coronary stenoses. Circ Cardiovasc Interv. (2014) 7(1):35–42. 10.1161/CIRCINTERVENTIONS.113.000659 24399244

[127] Liu Y Ding L-Y Li X. Therapy with ticagrelor for ST-elevated acute coronary syndrome accompanied by diabetes mellitus. Eur Rev Med Pharmacol Sci. (2019) 23:312–8. 10.26355/eurrev_201908_18662 31389599

[128] Salvatore T Pafundi PC Morgillo F Di Liello R Galiero R Nevola R Metformin: an old drug against old age and associated morbidities. Diabetes Res Clin Pract. (2020) 160:108025. 10.1016/j.diabres.2020.108025 31954752

[129] van Leeuwen MA van der Hoeven NW Janssens GN Everaars H Nap A Lemkes JS Evaluation of microvascular injury in revascularized patients with ST-segment–elevation myocardial infarction treated with ticagrelor versus prasugrel: the REDUCE-MVI trial. Circulation. (2019) 139(5):636–46. 10.1161/CIRCULATIONAHA.118.035931 30586720

[130] Zhang H-T Jia ZH Zhang J Ye ZK Yang WX Tian YQ No-reflow protection and long-term efficacy for acute myocardial infarction with tongxinluo: a randomized double-blind placebo-controlled multicenter clinical trial (ENLEAT trial). Chin Med J. (2010) 123(20):2858–64. 21034597

[131] Ma Q Ma Y Wang X Li S Yu T Duan W Intracoronary compared with intravenous bolus tirofiban on the microvascular obstruction in patients with STEMI undergoing PCI: a cardiac MR study. Int J Cardiovasc Imaging. (2020) 36:1121–32. 10.1007/s10554-020-01800-0 32078096

[132] McCartney PJ Eteiba H Maznyczka AM McEntegart M Greenwood JP Muir DF Effect of low-dose intracoronary alteplase during primary percutaneous coronary intervention on microvascular obstruction in patients with acute myocardial infarction: a randomized clinical trial. JAMA. (2019) 321(1):56–68. 10.1001/jama.2018.19802 30620371 PMC6583564

[133] Niccoli G Rigattieri S De Vita MR Valgimigli M Corvo P Fabbiocchi F Open-label, randomized, placebo-controlled evaluation of intracoronary adenosine or nitroprusside after thrombus aspiration during primary percutaneous coronary intervention for the prevention of microvascular obstruction in acute myocardial infarction: the REOPEN-AMI study (intracoronary nitroprusside versus adenosine in acute myocardial infarction). JACC Cardiovasc Interv. (2013) 6(6):580–9. 10.1016/j.jcin.2013.02.009 23683738

[134] Qian G Zhang Y Dong W Jiang ZC Li T Cheng LQ Effects of nicorandil administration on infarct size in patients with ST-segment–elevation myocardial infarction undergoing primary percutaneous coronary intervention: the CHANGE trial. J Am Heart Assoc. (2022) 11(18):e026232. 10.1161/JAHA.122.026232 36073634 PMC9683654

[135] Zhao S Qi G Tian W Chen L Sun Y. Effect of intracoronary nitroprusside in preventing no reflow phenomenon during primary percutaneous coronary intervention: a meta-analysis. J Interv Cardiol. (2014) 27(4):356–64. 10.1111/joic.12133 25041036

[136] Huang D Qian J Ge L Jin X Jin H Ma J REstoration of COronary flow in patients with no-reflow after primary coronary interVEntion of acute myocaRdial infarction (RECOVER). Am Heart J. (2012) 164(3):394–401. 10.1016/j.ahj.2012.06.015 22980307

[137] Jolly SS James S Džavík V Cairns JA Mahmoud KD Zijlstra F Thrombus aspiration in ST-segment–elevation myocardial infarction: an individual patient meta-analysis: thrombectomy trialists collaboration. Circulation. (2017) 135(2):143–52. 10.1161/CIRCULATIONAHA.116.025371 27941066

[138] Shibata N Takagi K Morishima I Yoshioka N Furui K Nagai H The impact of the excimer laser on myocardial salvage in ST-elevation acute myocardial infarction via nuclear scintigraphy. Int J Cardiovasc Imaging. (2020) 36:161–70. 10.1007/s10554-019-01690-x 31451993

[139] Carrick D Oldroyd KG McEntegart M Haig C Petrie MC Eteiba H A randomized trial of deferred stenting versus immediate stenting to prevent no-or slow-reflow in acute ST-segment elevation myocardial infarction (DEFER-STEMI). J Am Coll Cardiol. (2014) 63(20):2088–98. 10.1016/j.jacc.2014.02.530 24583294 PMC4029071

[140] Pompei G Ganzorig N Kotanidis CP Alkhalil M Collet C Sinha A Novel diagnostic approaches and management of coronary microvascular dysfunction. Am J Prev Cardiol. (2024) 19:100712. 10.1016/j.ajpc.2024.100712 39161975 PMC11332818

[141] Chan W Taylor AJ Ellims AH Lefkovits L Wong C Kingwell BA Effect of iron chelation on myocardial infarct size and oxidative stress in ST-elevation–myocardial infarction. Circ Cardiovasc Interv. (2012) 5(2):270–8. 10.1161/CIRCINTERVENTIONS.111.966226 22496085

[142] García-Prieto J Villena-Gutiérrez R Gómez M Bernardo E Pun-García A García-Lunar I Neutrophil stunning by metoprolol reduces infarct size. Nat Commun. (2017) 8:14780. 10.1038/ncomms14780 28416795 PMC5399300

[143] Roolvink V Ibáñez B Ottervanger JP Pizarro G van Royen N Mateos A Early intravenous beta-blockers in patients with ST-segment elevation myocardial infarction before primary percutaneous coronary intervention. J Am Coll Cardiol. (2016) 67(23):2705–15. 10.1016/j.jacc.2016.03.522 27050189

[144] Zhang H Tian NL Hu ZY Wang F Chen L Zhang YJ Three hours continuous injection of adenosine improved left ventricular function and infarct size in patients with ST-segment elevation myocardial infarction. Chin Med J. (2012) 125(10):1713–9. 22800889

[145] Zhou J Xu J Cheng A Li P Chen B Sun S. Effect of nicorandil treatment adjunctive to percutaneous coronary intervention in patients with acute myocardial infarction: a systematic review and meta-analysis. J Int Med Res. (2020) 48(11):0300060520967856. 10.1177/0300060520967856 33249959 PMC7708727

[146] Chen W Spitzl A Mathes D Nikolaev VO Werner F Weirather J Endothelial actions of ANP enhance myocardial inflammatory infiltration in the early phase after acute infarction. Circ Res. (2016) 119(2):237–48. 10.1161/CIRCRESAHA.115.307196 27142162

[147] Kitakaze M Asakura M Kim J Shintani Y Asanuma H Hamasaki T Human atrial natriuretic peptide and nicorandil as adjuncts to reperfusion treatment for acute myocardial infarction (J-WIND): two randomised trials. Lancet. (2007) 370(9597):1483–93. 10.1016/S0140-6736(07)61634-1 17964349

[148] Vilahur G Gutiérrez M Casani L Varela L Capdevila A Pons-Lladó G Protective effects of ticagrelor on myocardial injury after infarction. Circulation. (2016) 134(22):1708–19. 10.1161/CIRCULATIONAHA.116.024014 27789556

[149] Van't Hof AW Ten Berg J Heestermans T Dill T Funck RC van Werkum W Prehospital initiation of tirofiban in patients with ST-elevation myocardial infarction undergoing primary angioplasty (on-TIME 2): a multicentre, double-blind, randomised controlled trial. Lancet. (2008) 372(9638):537–46. 10.1016/S0140-6736(08)61235-0 18707985

[150] Zhao ZZ Li E Li XJ Guo Q Shi QB Li MW. Effects of remote ischemic preconditioning on coronary blood flow and microcirculation. BMC Cardiovasc Disord. (2023) 23(1):404. 10.1186/s12872-023-03419-0 37592218 PMC10433538

[151] Lang JA Kim J. Remote ischaemic preconditioning—translating cardiovascular benefits to humans. J Physiol. (2022) 600(13):3053–67. 10.1113/JP282568 35596644 PMC9327506

[152] Sloth AD Schmidt MR Munk K Kharbanda RK Redington AN Schmidt M Improved long-term clinical outcomes in patients with ST-elevation myocardial infarction undergoing remote ischaemic conditioning as an adjunct to primary percutaneous coronary intervention. Eur Heart J. (2014) 35(3):168–75. 10.1093/eurheartj/eht369 24031025

[153] Wang M Shan Y Sun W Han J Tong H Fan M Effects of shexiang baoxin pill for coronary microvascular function: a systematic review and meta-analysis. Front Pharmacol. (2021) 12:751050. 10.3389/fphar.2021.751050 34795585 PMC8592925

[154] Yu YW Liu S Zhou YY Huang KY Wu BS Lin ZH Shexiang baoxin pill attenuates myocardial ischemia/reperfusion injury by activating autophagy via modulating the ceRNA-Map3k8 pathway. Phytomedicine. (2022) 104:154336. 10.1016/j.phymed.2022.154336 35849969

[155] Xu ZJ Dai XH Tang LF Huang HY Mao DM Zhou P. The effect of Qishen Yiqi Dripping Pills on cardiac function and angina pectoris in patients with coronary heart disease after PCI surgery. J Tradit Chin Med. (2019) 30(09):2208–9.

[156] Cheng L Meng GTY Liang JG. Myocardial Protection Effect of Qishen Yiqi Dropping pills on patients after PCI and control effect and mechanism on cardiac adverse events. Chin J Exp Tradit Med Formulae. (2019) 25(16):78–84.

[157] Li M Wang Y Qi Z Yuan Z Lv S Zheng Y Qishenyiqi dripping pill protects against myocardial ischemia/reperfusion injury via suppressing excessive autophagy and NLRP3 inflammasome based on network pharmacology and experimental pharmacology. Front Pharmacol. (2022) 13:981206. 10.3389/fphar.2022.981206 36164369 PMC9507923

[158] Shao J. Study on the therapeutic effect of Tongxinluo capsule on patients with coronary heart disease after interventional surgery and its impact on vascular endothelial function and inflammatory factors. Guizhou Med J. (2022) 46(02):294–6.

[159] Yan SY Ma LH Guo CX Lu PP Su YN Guo XD Prospective cohort study on improving the prognosis of patients with coronary heart disease after PCI. Zhongguo Zhong Xi Yi Jie He Za Zhi (2022) 42(11):1300–6.

